# Novel species of *Cladosporium* from environmental sources in Spain

**DOI:** 10.3897/mycokeys.77.60862

**Published:** 2021-01-05

**Authors:** Isabel Iturrieta-González, Dania García, Josepa Gené

**Affiliations:** 1 Unitat de Micologia, Facultat de Medicina i Ciències de la Salut and IISPV, Universitat Rovira i Virgili, 43201, Reus, Tarragona, Spain Universitat Rovira i Virgili Reus Spain

**Keywords:** *
Cladosporiales
*, *
Cladosporiaceae
*, hyphomycetes, phylogeny, Spain, taxonomy

## Abstract

*Cladosporium* is a monophyletic genus in *Cladosporiaceae* (*Cladosporiales*, *Dothideomycetes*) whose species are mainly found as saprobes and endophytes, but it also includes fungi pathogenic for plants, animals and human. Species identification is currently based on three genetic markers, viz., the internal transcribed spacer regions (ITS) of the rDNA, and partial fragments of actin (*act*) and the translation elongation factor 1-α (*tef1*) genes. Using this phylogenetic approach and from morphological differences, we have recognized six new species originating from soil, herbivore dung and plant material collected at different Spanish locations. They are proposed as *Cladosporium caprifimosum*, *C. coprophilum*, *C. fuscoviride* and *C. lentulum* belonging in the *C. cladosporioides* species complex, and *C. pseudotenellum* and *C. submersum* belonging in the *C. herbarum* species complex. This study revealed that herbivore dung represented a reservoir of novel lineages in the genus *Cladosporium*.

## Introduction

*Cladosporium* is a ubiquitous genus in the family *Cladosporiaceae* of the recently proposed order *Cladosporiales* in the *Dothideomycetes* (Abdollahzadeh et al. 2020). Their species inhabit a wide range of substrates and have been reported to be among the most common fungi in both indoor and outdoor environments, including in extreme ecological niches (Flannigan et al. 2002; Bensch et al. 2010, 2012, 2018; Sandoval-Denis et al. 2015; Temperini et al. 2018; Chung et al. 2019). Most *Cladosporium* species are saprobic, but some have also been reported as endophytes, hyperparasites on other fungi and plant as well as animal pathogens, including humans (Heuchert et al. 2005; Sandoval-Denis et al. 2016; de Hoog et al. 2017; Marin-Felix et al. 2017). Certain species show the ability to produce compounds of medical interest or are relevant as potential biocontrol agents for plant disease (Köhl et al. 2015; Khan et al. 2016; Adorisio et al. 2019).

*Cladosporium* is morphologically characterized mainly by its asexual morph, which shows differentiated conidiophores producing acropetal chains of conidia from mono- or polyblastic conidiogenous cells. Both conidiogenous cells and conidia exhibit conidiogenous loci (scars) with a unique coronate structure, which is composed of a central convex dome surrounded by a raised periclinal rim, usually thickened, refractive and darkly pigmented (David 1997). Based on these features and DNA phylogeny derived from the LSU nrRNA gene, the genus has been well-delineated and distinguished from other cladosporium-like genera such as *Hyalodendriella*, *Ochrocladosporium*, *Rachicladosporium*, *Rhizocladosporium*, *Toxicocladosporium*, *Verrucocladosporium* and the recently described genus *Neocladosporium* (Crous et al. 2007; Bezerra et al. 2017). Phylogenetic relationships among species of *Cladosporiums. str.* have been studied extensively over the last decade by a multi-locus sequence analysis approach with sequences of the internal transcribed spacers (ITS) of the rDNA and of the two protein encoding genes, translation elongation factor 1-α (*tef1*) and actin (*act*). The molecular approach combined with morphological features have allowed recognition of more than 230 species within the genus, which are split into three species complexes, i.e., the *Cladosporium cladosporioides*, *Cladosporium herbarum* and *Cladosporium sphaerospermum* complex (Schubert et al. 2007; Bensch et al. 2010, 2012, 2015, 2018; Sandoval-Denis et al. 2016; Marin-Felix et al. 2017).

While aiming to explore the diversity of microfungi from Spain, several interesting *Cladosporium* isolates have been recovered from different environmental samples. Using the above mentioned polyphasic approach and following the Genealogical Phylogenetic Species Recognition (GCPSR) criterion (Taylor et al. 2000), the taxonomy of those isolates has been resolved in six novel species for science; four pertaining to the *C. cladosporioides* species complex and two to the *C. herbarum* complex.

## Material and methods

### Samples and isolates

Samples of soil, plant debris and herbivore dung were collected between 2016 and 2018 at various Spanish locations. Dilution plating methods were used for isolating fungi from soil and dung samples following the procedure described by Crous et al. (2009) and a modified protocol described by Waksman (1922), respectively. In addition, soil samples were also processed by a baiting technique using small pieces of wood and filter paper as baits on the soil surface (Calduch et al. 2004). Samples of plant debris and also part of the herbivore dung were incubated in moist chambers following the procedures described by Castañeda-Ruiz et al. (2016) and Richardson (2001), respectively.

Among the cladosporium-like fungi found, we recovered eight isolates in pure culture which did not match any of the currently accepted species within the genus *Cladosporium* (Table 1). The isolates were deposited in the culture collection of the Universitat Rovira i Virgili (FMR, Reus, Spain) and, once phylogenetically and morphologically characterized, living cultures of the novel species and dry cultures for holotypes were also deposited in the Westerdijk Fungal Biodiversity Institute (CBS; Utrecht, the Netherlands). Nomenclatural novelties and descriptions were deposited in MycoBank (Crous et al. 2004).

**Table 1.** *Cladosporium* species, strain information and GenBank accession numbers for sequences obtained in this study.

**Species**	**Strain number^1^**	**Substrate**	**GenBank nucleotide accession no. for^2^**:		
			**ITS**	***act***	***tef1***
*C. caprifimosum*	FMR 16532^T^	Goat dung	LR813198	LR813205	LR813210
*C. coprophilum*	FMR 16101	Unidentified herbivore dung	LR813199	LR813204	LR813211
	FMR 16164^T^	Unidentified herbivore dung	LR813201	LR813207	LR813213
*C. fuscoviride*	FMR 16385^T^	Garden soil	LR813200	LR813206	LR813212
*C. lentulum*	FMR 16288^T^	Unidentified leaf litter	LR813203	LR813209	LR813215
	FMR 16389	Unidentified herbivore dung	LR813202	LR813208	LR813214
*C. pseudotenellum*	FMR 16231^T^	Garden soil	LR813145	LR813146	LR813196
*C. submersum*	FMR 17264^T^	Submerged plant material	LR813144	LR813195	LR813197

^1^FMR: Facultat de Medicina i Ciències de la Salut, Reus, Spain. ^T^ indicate ex-type strains. ^2^ITS: Internal transcribed spacer regions of the rDNA and 5.8S region; *act*: partial actin gene; *tef1*: partial translation elongation factor 1-alpha gene.

### DNA extraction, amplification and sequencing

Genomic DNA was extracted from cultures growing on potato dextrose agar (PDA; Pronadisa, Spain) after 7 days of incubation at 25 °C, following the modified protocol of Müller et al. (1998). Protocols listed previously in Sandoval-Denis et al. (2016) were used for amplification and sequencing. The primer pairs used were ITS5/ITS4 (White et al. 1990) to amplify the ITS region including the 5.8S gene of the rDNA, EF-728F/EF-986R to amplify a partial fragment of the *tef1* gene, and ACT-512F/ACT-783R to amplify a partial fragment of *act* gene (Carbone and Kohn 1999). PCR products were purified and stored at -20 °C until sequencing. The sequences were obtained using the same primers at Macrogen Europe (Macrogen Inc. Amsterdam, The Netherlands). Finally, the software SeqMan v. 7.0.0 (DNAStarLasergene, Madison, WI, USA) was used to assemble, edit and obtain the consensus sequences, which were then deposited in GenBank of the National Center for Biotechnology Information (NCBI) (Table 1).

### Sequence alignment and phylogenetic analysis

The sequences obtained were compared with other fungal sequences deposited in the NCBI database through the BLASTn tool. Alignment of those sequences and the phylogenetic analysis for each locus were performed with the MEGA (Molecular Evolutionary Genetics Analysis) program v. 6.0. (Tamura et al. 2013), using ClustalW algorithm (Thompson et al. 1994) and refined with MUSCLE (Edgar 2004) or manually if necessary, on the same platform. Since the isolates under study were related to the *C. cladosporioides* and *C. herbarum* species complexes, we also carried out alignments including sequence data of ex-type and reference strains of all the species from both complexes retrieved from the GenBank and mainly published by Schubert et al. (2007, 2009), Bensch et al. (2010, 2012, 2015, 2018), Sandoval-Denis et al. (2016) and Marin-Felix et al. (2017) (Suppl. material 1: Table S1).

Phylogenetic reconstructions were made with the three phylogenetic markers (ITS, *act* and *tef1*) recommended for an accurate identification at the species level (Bensch et al. 2010, 2018; Marin-Felix et al. 2017) using Maximum Likelihood (ML), Maximum Parsimony (MP), and Bayesian Inference (BI) analyses, with the Mega software v. 6.0. for the former two (Tamura et al. 2013) and with MrBayes v.3.2.6 for the latter one (Ronquist et al. 2012). Phylogenetic concordance of the three-locus datasets was evaluated through Incongruence Length Difference (ILD) implemented in the Winclada program (Farris et al. 1994) and also by visual comparison of the individual phylogenies in order to assess any incongruent results between nodes with high statistical support.

Determined by Mega software v. 6.0., the best nucleotide substitution model for ML analysis of the *C. cladosporioides* complex was General Time Reversible with Gamma distribution and invariant sites (GTR+G+I), and for the *C. herbarum* complex the best was the Kimura 2-parameter with Gamma distribution and invariant sites (K2+G+I). Bootstrap support value (MLBS) ≥ 70% was considered significant (Hillis and Bull 1993).

The MP analysis was performed using the heuristic search option with TBR (tree bisection and reconnection) branch swapping and 1,000 random sequence additions. Tree length (TL), consistency index (CI), retention index (RI), rescaled consistency index (RCI) were calculated. Bootstrap analysis was based on 1,000 replications. Maximum parsimony bootstrap support value (PBS) ≥ 70% was considered significant (Hillis and Bull 1993).

Determined by jModelTest (Posada 2008), the best nucleotide substitution models for the BI of the *C. cladosporioides* complex were Jukes Cantor with invariant sites (JC+I) for ITS, General Time Reversible with Gamma distribution (GTR+G) for *tef1* and Hasegawa-Kishino-Yano with Gamma distribution (HKY+G) for *act*; and for the *C. herbarum* complex the best were the Kimura 2-parameter with Gamma distribution (K80+G) for ITS, Hasegawa-Kishino-Yano with Gamma distribution (HKY+G) for *tef1* and *act.* The parameter settings used in these analyses were two simultaneous runs of 10,000,000 generations, and four Markov chains, sampled every 1,000 generations. The 50% majority rule consensus tree and posterior probability values (PP) were calculated after discarding the first 25% of the samples. A PP value of ≥ 0.95 was considered significant (Hespanhol et al. 2019).

Final sequence alignments and trees generated in this study were registered in TreeBASE under the submission number S27350 (http://treebase.org).

### Phenotypic studies

Microscopic features of the *Cladosporium* isolates were obtained from cultures growing on synthetic nutrient-poor agar (SNA; 1 g of KH_2_PO_4_, 1 g of KNO_3_, 0.5 g of MgSO_4_ × 7H_2_O, 0.5 g of KCl, 0.2 g of glucose, 0.2 g of sucrose, 14 g of bacteriological agar, 1 L of distilled water) after 7 to 14 days at 25 °C in the dark, mounted onto semi-permanent slides with Shear's solution (Bensch et al. 2018). At least 30 measurements were taken to calculate length and width ranges of the conidia and ramoconidia, given as the mean ± standard deviation in the descriptions. Macroscopic characterization of the colonies was made on PDA (Pronadisa, Spain), oatmeal agar (OA; 30 g of oatmeal, 13 g of bacteriological agar, 1 L distilled water) and SNA after 14 days of incubation at 25 °C in darkness. Colour notation of the colonies in descriptions were from Kornerup and Wanscher (1978). In addition, cardinal temperatures for the fungal growth were determined on PDA cultures after 14 days at temperatures ranging from 5 to 40 °C at intervals of 5 °C.

## Results

### Phylogeny

Three individual phylogenies (ITS, *tef1* and *act*), carried out for the *C. cladosporioides* and *C. herbarum* species complexes, were visually very similar and the ILD test showed that the three loci datasets were congruent in both complexes (*P* = 0.16) and could be combined. Phylogenies obtained by ML, MP and BI also showed a visual topological congruence and were similar to that obtained by other authors (Marin-Felix et al. 2017; Bensch et al. 2018). The combined alignment of the three mentioned loci datasets encompassed 101 sequences in the *C. cladosporioides* complex and 58 sequences in *C. herbarum* complex. The alignment for the former group comprised 1,060 bp (ITS 484 bp, *tef1* 313 bp and *act* 263 bp), which included 424 bp variable sites (ITS 47 bp, *tef1* 239 bp and *act* 138 bp) and 319 bp phylogenetically informative sites (ITS 25 bp, *tef1* 193 bp and *act* 101 bp). Two species of the *C. sphaerospermum* complex, *C. sphaerospermum*CBS 193.54 and *C. longissimum*CBS 300.96, were included as outgroup in this first multi-locus phylogeny (Fig. 1). For the maximum parsimony analysis the maximum of 1,000 equally most parsimonious trees were saved (Tree length = 1614; CI = 0.294; RI = 0.666; RCI = 0.214).

**Figure 1.**
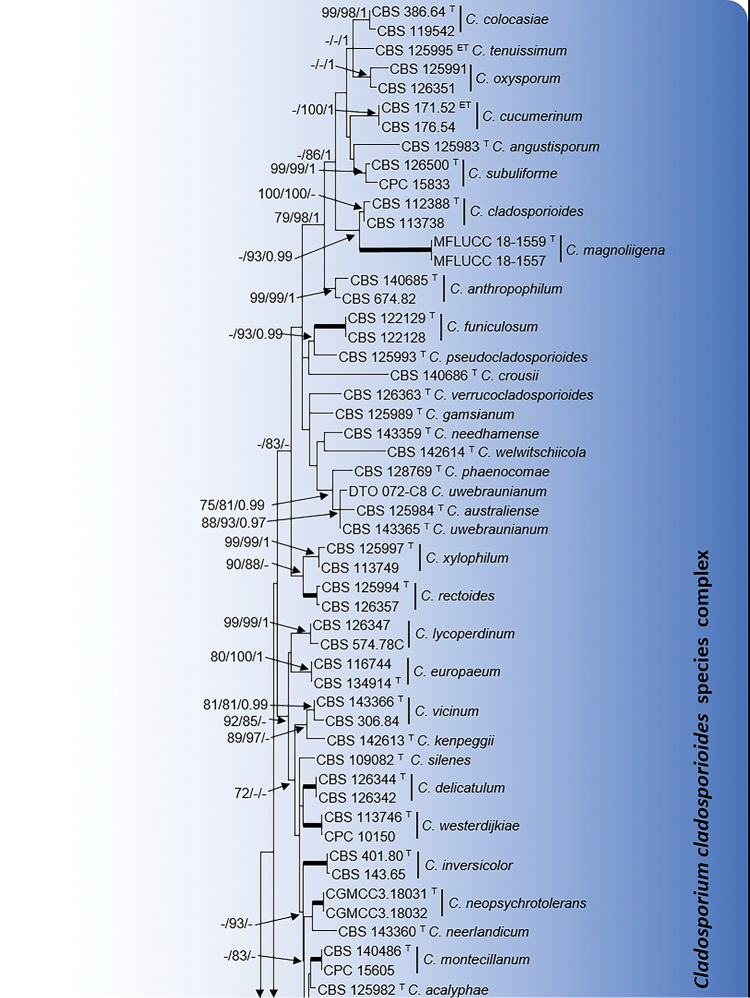
Maximum likelihood (ML) tree obtained from the combined analysis of ITS, *tef1* and *act* sequences of 101 strains from the *C. cladosporioides* complex. The tree is rooted with *C. sphaerospermum*CBS 193.54 and *C. longissimum*CBS 300.96. Numbers on the branches represent ML bootstrap support values (MLBS) ≥70%, followed by Maximum Parsimony bootstrap support values (PBS) ≥70% and Bayesian posterior probabilities (PP) ≥0.95, lower values are indicate as “-“. Bold branches indicate MLBS/PBS/PP of 100/100/1. Names of species newly described are indicated in bold. Branch lengths are proportional to distance. ^T^ Ex-type strain. ^ET^ Ex-epitype strain.

**Figure 1.**
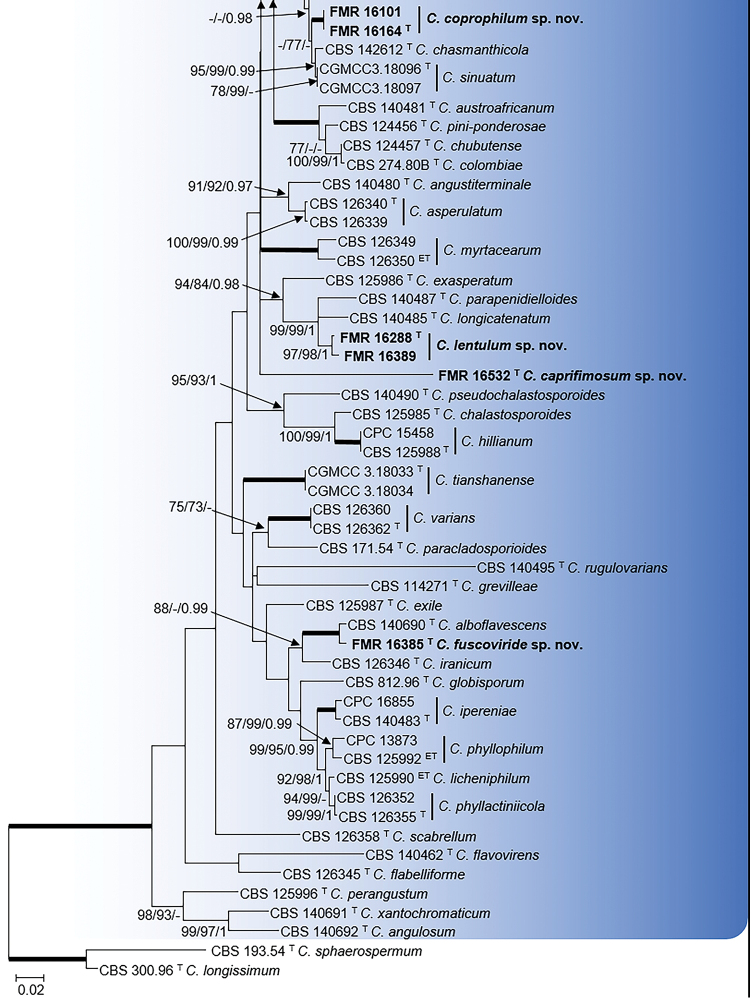
Continued.

**Figure 2.**
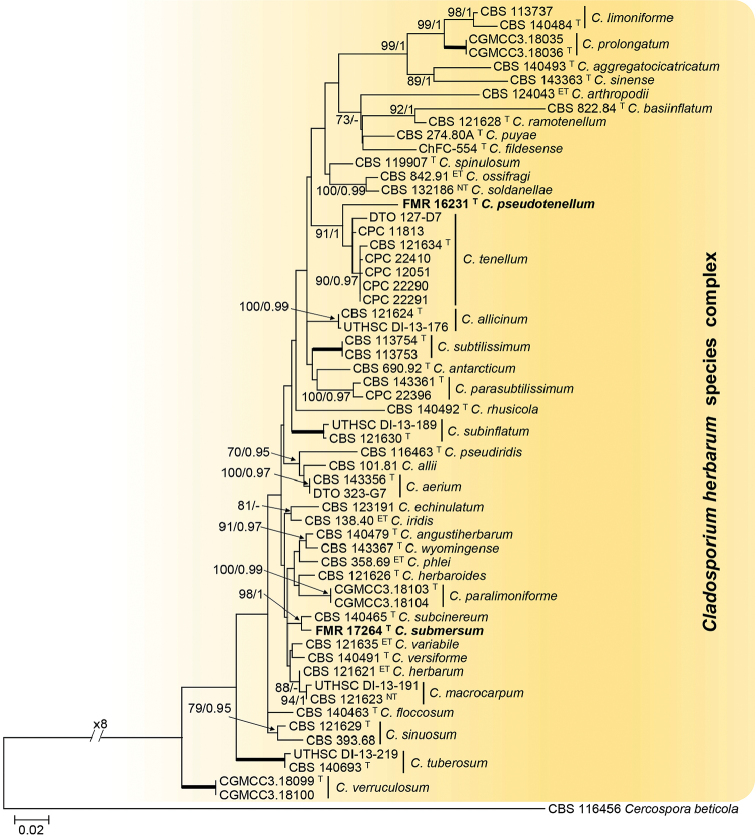
Maximum likelihood (ML) tree obtained from the combined analysis of ITS, *tef1* and *act* sequences of 58 strains from *C. herbarum* complex. The tree is rooted with *Cercospora beticola*CBS 116456. Numbers on the branches represent ML bootstrap support values (MLBS) ≥70%, followed by Maximum Parsimony bootstrap support values (PBS) ≥70% and Bayesian posterior probabilities (PP) ≥ 0.95, lower values are indicate as “-“. Bold branches indicate MLBS/PBS/PP of 100/100/1. Names of species newly described are indicated in bold. Branch lengths are proportional to distance. ^T^ Ex-type strain. ^ET^ ex-epitype strain. ^NT^ ex-neotype strain.

For the *C. herbarum* species complex, the alignment comprised 1,057 bp (ITS 503 bp, *tef1* 309 bp and *act* 245 bp) with 407 bp variable sites (ITS 101 bp, *tef1* 186 bp and *act* 120 bp) and 240 bp phylogenetically informative sites (ITS 27 bp, *tef1* 123 bp and *act* 90 bp), using *Cercospora beticola* (CBS 116456) as outgroup (Fig. 2). For the maximum parsimony analysis the maximum of 1,000 equally most parsimonious trees were saved (Tree length = 898; CI = 0.537; RI = 0.661; RCI = 0.355).

The eight unidentified isolates did not match any known lineage of *Cladosporium* species, six were related to the *C. cladosporioides* species complex and two to the *C. herbarum* complex, and together they represented six new phylogenetic species in the genus.

In the combined phylogeny of the *C. cladosporioides* complex, 71 species were delineated (Fig. 1). The isolates FMR 16101 and FMR 16164 formed a strongly supported terminal clade representative for a unique taxon, but with an uncertain phylogenetic position due to the low statistical support (- MLBS / 77 PBS / - PP) for the nearest lineages of *C. chasmanthicola* and *C. sinuatum*. Both unidentified isolates were genetically identical and showed a percentage of identity with the ex-type strains of these latter species of 97.22% and 97.65% for *act*, and 96.79% and 97.50% for *tef1*, respectively. A second undescribed monophyletic terminal clade included FMR 16288 and FMR 16389, which grouped with the lineages of *C. exasperatum*, *C. parapenidielloides* and *C. longicatenatum* in a clade with highly supported values (94 MLBS / 84 PBS / 0.98 PP). However, both isolates showed a sufficient genetic distance to be considered a distinct species from the closest, *C. longicatenatum* and *C. parapenidielloides*, with a sequence similarity of 95.75% and 95.28% for *act* and 90.87% and 90.48% for *tef1* respect to the ex-type strains of these two known species. FMR 16532 and FMR 16385 formed two distinct monophyletic branches. The former showed an uncertain phylogenetic position with the species in the complex; the comparison of its sequences with those of the GenBank dataset through the BLASTn tool showed that the ITS was 100% similar with several species of the *C. cladosporioides* complex, while sequences of *act* and *tef1* were 99.12% and 89.02% similar with sequences belonging to *C. asperulatum* (UTHSC DI-13-216/GenBank LN834541 and CBS 113744/GenBank HM148237, respectively). FMR 16385 was closely related to the ex-type strain of *C. alboflavescens* (100 MLBS / 100 PBS / 1 PP). The percentages of identity between these latter two fungi (97.79% for *act* and 96.75% for *tef1*) together with morphological differences observed allow us to consider them distinct taxa.

In the *C. herbarum* complex, 40 species were phylogenetically well-delimited, including two novel lineages each represented by FMR 16231 and FMR 17264 (Fig. 2). Both were genetically and morphologically differentiated from their closest relatives, *C. tenellum* and *C. subcinereum*, respectively. The percentages of identity observed between the isolate FMR 16231 and the ex-type strain of *C. tenellum* (CBS 121634) were 97.78%, 83.76% and 100% for *act*, *tef1* and ITS, respectively, and between FMR 17264 and the ex-type strain of *C. subcinereum* (CBS 140465) were 98.57%, 95.98% and 100% for *act*, *tef1* and ITS, respectively.

The percentages of identity between the six putative new *Cladosporium* species and their relatives are summarized in Table 2. The novel taxa are described and illustrated in the taxonomy section below.

**Table 2.** Percentage of identity between the novel *Cladosporium* and their closest species.

Species	Closest taxa	Loci		
		ITS	*act*	*tef1*
*C. caprifimosum* (FMR 16532)	*C. asperulatum* ^1^	100	99.12	89.02
*C. coprophilum* (FMR 16101 and 16164)	*C. chasmanthicola*	100	97.22	96.79
	*C. sinuatum* ^1^	100	97.65	97.50
*C. fuscoviride* (FMR 16385)	*C. alboflavescens*	100	97.79	96.75
*C. lentulum* (FMR 16288 and 16389)	*C. exasperatum*	100	92.2	81.4–82.7
	*C. longicatenatum*	100	95.75	90.87
	*C. parapenidielloides*	100	95.28	90.48
*C. pseudotenellum* (FMR 16231)	*C. tenellum* ^1^	100	97.5–100	83.2–84.1
*C. submersum* (FMR 17264)	*C. subcinereum*	100	98.57	95.98

^1^ Species for which the percentage of identity was defined based on the ex-type strain and additional reference strains (see Figs 1 and 2). ^2^ Species for which the percentage of identity was based on a NCBI BLAST search.

## Taxonomy

### Cladosporium caprifimosum

Taxon classificationFungi

Iturrieta-González, Dania García, Gené sp. nov.

F169FB60-1BAE-50F8-BC05-A0BC0DBCB16A

836074

Fig. 3

#### Etymology.

The name refers to goat dung, the substrate where the species was isolated (capra = goat and fimus = dung, with the adjectival Latin suffix -osus, indicating abundance or full or marked development).

#### Type.

Spain, Catalonia, Tarragona province, La Fatarella, from goat dung, Mar. 2017, *I. Iturrieta-González, M. Guevara-Suarez & J. Guarro* (holotype CBS H-24469; cultures ex-type FMR 16532, CBS 146918).

#### Description.

*Mycelium* in vitro superficial and immersed, composed of septate, branched, subhyaline, smooth to verruculose hyphae, 1–2 μm wide. *Conidiophores* dimorphic, micronematous or macronematous, arising from lateral or terminal hyphae, erect to slightly flexuous, non-nodulose, septate, branched or unbranched, 8–137 μm long, 2–4 μm wide, pale brown, slightly verrucose. *Conidiogenous cells* integrated, terminal, cylindrical, sometimes geniculate at the apex, 22–44 × 3–4 μm, bearing up to four conidiogenous loci, darkened and refractive. *Ramoconidia* aseptate, almost cylindrical, 10–24 × 2–4 μm [av. (± SD) 15.8 (± 3.4) × 3.1 (± 0.45)], olive to pale brown, smooth. *Conidia* forming branched chains, with up to five conidia in the terminal unbranched part, aseptate, olive to pale brown, smooth; *small terminal conidia* ellipsoidal to obovoid, 3–7 × 2–3.5 μm [av. (± SD) 5.7 (± 0.83) × 2.4 (± 0.43)]; *intercalary conidia* ellipsoidal to somewhat fusiform, 6–11.5 × 2–3 μm [av. (± SD) 7.8 (± 1.06) × 2.6 (± 0.39)]; *secondary ramoconidia* ellipsoidal to almost cylindrical, 9–14 × 2.5–3.5 μm [av. (± SD) 11.3 (± 1.6) × 2.9 (± 0.26)].

#### Culture characteristics

(14 d at 25 °C). Colonies on OA reaching 24–25 mm diam., dark green (30F8), flat, slightly dusty, aerial mycelium scarce, margin regular; reverse dark green (30F8) to black. On PDA attaining 34–35 mm diam., olive (3E6/3F4), slightly umbonate, radially folded, velvety, aerial mycelium scarce, margin slightly lobate; reverse dark green (30F8) to olive (3E4). On SNA reaching 25–26 mm diam., olive (3E8), flat, dusty, aerial mycelium scarce, margin regular; reverse dark green (30F8) to black.

#### Cardinal temperature for growth.

Optimum 20 °C, maximum 30 °C, minimum 5 °C.

#### Distribution.

Spain.

#### Notes.

Although *C. caprifimosum* clearly belongs to the *C. cladosporioides* species complex, our multi-locus analysis did not reveal any phylogenetic relationship with other species in the complex. It is represented by a single branch placed distance from other *Cladosporium* species (Fig. 1). *Cladosporium caprifimosum* differs from the other novel species proposed here mainly by its aseptate and smooth conidia.

**Figure 3.**
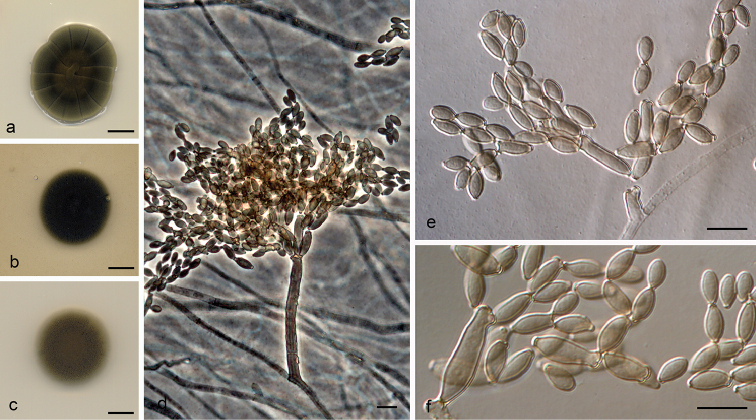
*Cladosporium caprifimosum* (ex-type FMR 16532) **a–c** colonies on PDA, OA and SNA after 14 days at 25 °C **d–e** conidiophores **f** ramoconidia and conidia. Scale bars: 10 mm (**a–c**); 10 μm (**d–f**).

### Cladosporium coprophilum

Taxon classificationFungi

Iturrieta-González, Dania García, Gené sp. nov.

2450FD97-632B-5CF8-B421-283EC5582623

836075

Fig. 4

#### Etymology.

Name refers to the substrate where the species was isolated, unidentified herbivore dung (ancient Greek, kópros = dung + phílos = loving).

#### Type.

Spain, Extremadura, Badajoz province, Granja de Torrehermosa, unidentified herbivore dung, Jan. 2017, *J. Cano* (holotype CBS H-24470; cultures ex-type FMR 16164, CBS 144919).

#### Description.

*Mycelium* in vitro superficial and immersed, composed of septate, branched, pale brown, smooth hyphae, 3–5 μm wide. *Conidiophores* macronematous, arising laterally or terminally from hyphae, erect to slightly flexuous, non-nodulose, septate, unbranched, up to 124 μm long, 3–4 μm wide, pale brown, smooth. *Conidiogenous cells* integrated, terminal, rarely intercalary, cylindrical, (7–)14–33 × (2–)3–4 μm, bearing up to 3 conidiogenous loci, slightly darkened and refractive. *Ramoconidia* 0(–1)-septate, subcylindrical to cylindrical, 9–19 × 3–5 μm [av. (± SD) 12.3 (± 2.8) × 3.9 (± 0.54)], pale brown, smooth. *Conidia* forming branched chains, with up to five conidia in the terminal unbranched part, aseptate, pale brown, smooth to verruculose; *small terminal conidia* ellipsoidal to slightly obovoid, 4.5–7 × 2.5–4 μm [av. (± SD) 6 (± 0.64) × 3.1 (± 0.31)]; *intercalary conidia* ellipsoidal, 6–10.5 × 2.5–4 μm [av. (± SD) 7.7 (± 1.32) × 3.3 (± 0.37)]; *secondary ramoconidia* subcylindrical to cylindrical, 7–12.5 μm long × 3–5 μm [av. (± SD) 9.6 (± 1.7) × 4.2 (± 0.51)].

#### Culture characteristics

(14 d at 25 °C). Colonies on OA reaching 21–22 mm diam., olive (2F6) to black, dark green margin (30F4), flat, slightly dusty at the center, aerial mycelium scarce, margin regular; reverse dark green (30F8) to black. On PDA attaining 36–37 mm diam., olive (2F6/2E3), greenish gray margin, slightly depressed and irregularly folded at the center, velvety, aerial mycelium scarce, margin regular; reverse dark green (30F8/27F3). On SNA reaching 27–28 mm diam., olive (3F6/2F8), flat, slightly dusty, aerial mycelium scarce, margin regular; reverse dark green (30F8) to black.

#### Cardinal temperature for growth.

Optimum 20 °C, maximum 25 °C, minimum 5 °C.

#### Distribution.

Spain.

#### Additional specimen examined.

Spain, Extremadura, Badajoz province, Granja de Torrehermosa, unidentified herbivore dung, Mar. 2017, *J. Cano* (FMR 16101).

#### Notes.

Based on the multi-locus analysis (Fig. 1), *C. coprophilum* is allocated to a terminal low-supported clade together with *C. chasmanthicola* and *C. sinuatum*, species recently described from leaf spots of *Chasmanthe aethiopica* in South Africa (Marin-Felix et al. 2017) and Alpine soil in China (Ma et al. 2017), respectively. The new species is distinguished from *C. chasmanthicola* by the production of smooth hyphae (smooth to distinctly verrucose or irregularly rough-walled in *C. chasmanthicola*), longer conidiogenous cells (up to 33 vs up to 24 μm), shorter ramoconidia (9–19 vs 15–33 μm) with fewer septa [(0(–1) vs 0–1(–3)-septate], and longer terminal conidia (4.5–7 vs 2.5–4.5 μm) (Marin-Felix et al. 2017). *Cladosporium coprophilum* differs from *C. sinuatum* by the production of aseptate intercalary conidia (0–1-septate in *C. sinuatum*). In addition, *C. sinuatum* is characterized by distinctive geniculate-sinuous conidiophores and a rather fast growth on OA (40–45 mm vs 21–22 mm in *C. coprophilum* after 14 d at 25 °C) (Ma et al. 2017).

**Figure 4.**
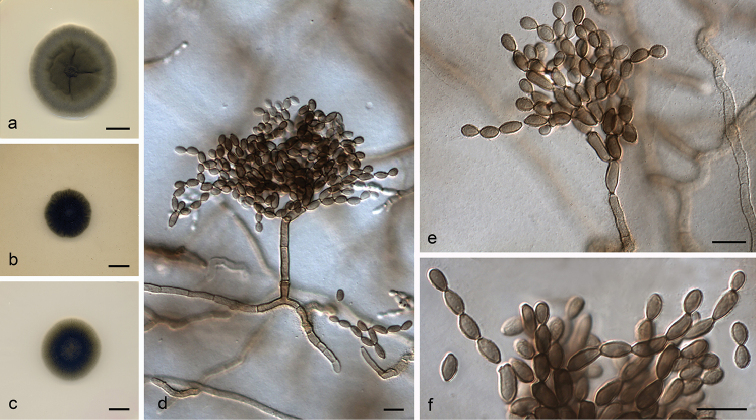
*Cladosporium coprophilum* (ex-type FMR 16164) **a–c** colonies on PDA, OA and SNA after 14 days at 25 °C **d–e** conidiophores **f** conidia. Scale bars: 10 mm (**a–c**); 10 μm (**d–f**).

### Cladosporium fuscoviride

Taxon classificationFungi

Iturrieta-González, Dania García, Gené sp. nov.

EB67E815-1B02-509A-84AA-1E26F800093F

836076

Fig. 5

#### Etymology.

Name refers to the dark green reverse of the colonies of the species growing in all agar media tested (fuscus = dark brown, blackish or figuratively dull and viridis = green).

#### Type.

Spain, Catalonia, Tarragona province, Cambrils, Samà Park, garden soil, Feb. 2017, *I. Iturrieta-González* & *J. Gené* (holotype CBS H-24471; cultures ex-type FMR 16385, CBS 146920).

#### Description.

*Mycelium* in vitro superficial and immersed, composed of septate, branched, subhyaline to pale brown, smooth to verruculose hyphae, 1–3 μm wide. *Conidiophores* semi-macronematous to macronematous, arising laterally and terminally from hyphae, sometimes reduced to conidiogenous cells, septate, erect to slightly flexuous, branched or unbranched, sometimes geniculate at the apex, up to 56 μm long, 3–4 μm wide, pale brown, smooth to verruculose. *Conidiogenous cells* terminal and subterminal, cylindrical to slightly clavate, 8–27 × 3–4 μm, bearing up to 4 conidiogenous loci, darkened and refractive. *Ramoconidia* 0–1(–3)-septate, subcylindrical to ellipsoidal, 7.5–22 × 2.5–4 μm [av. (± SD) 12.8 (± 3.9) × 3 (± 0.43)], pale brown, smooth to verruculose. *Conidia* in branched chains with up to 4 conidia in the terminal unbranched part, pale brown, smooth to verruculose, with protuberant, slightly darkened and refractive hila; *small terminal conidia* aseptate, globose, subglobose to obovoid, 3–6 × 2–3.5 μm [av. (± SD) 4.5 (± 0.66) × 3 (± 0.39)]; *intercalary conidia* aseptate, ellipsoidal to somewhat limoniform, 4.5–7 × 2.5–4 μm [av. (± SD) 5.7 (± 0.70) × 3.2 (± 0.36)]; *secondary ramoconidia* 0(–1)-septate, subcylindrical to ellipsoidal 6–11.5 × 2.5–4 μm [av. (± SD) 8.8 (± 1.64) × 3.1 (± 0.40)].

#### Culture characteristics

(14 d at 25 °C). Colonies on OA reaching 31–32 mm diam., olive (3F8) to dark green (30F5), olive final edge (2F8), flat, velvety, aerial mycelium scarce, margin regular; reverse dark green (30F5) to black. On PDA attaining 44–46 mm diam., gray to olive to olive yellow (3D1/2E5/2C6), white at the final edge, flat, velvety, aerial mycelium scarce, margin regular; reverse dark green (30F8) to black, with a whitish final edge. On SNA reaching 34–35 mm diam., olive (3F8), flat, velvety, aerial mycelium scarce, margin regular; reverse dark green (30F8), olive final edge (3F3).

#### Cardinal temperature for growth.

Optimum 25 °C, maximum 30 °C, minimum 5 °C.

#### Distribution.

Spain.

#### Notes.

*Cladosporium fuscoviride* is closely related to *C. alboflavescens* (Fig. 1), a monotypic species described from an animal respiratory specimen collected in California (Sandoval-Denis et al. 2016). The species can be distinguished by their colony and microscopic features; i.e., *C. fuscoviride* has darker colonies and faster growth rates at 25 °C after 2 wk on the three media tested (OA, 31–32 vs 20–23 mm; PDA, 44–46 vs 34–36 mm; SNA, 34–35 vs 20–25 mm), shorter conidiophores (up to 56 μm vs up to 130 μm long in *C. alboflavescens*), and 0–3-septate (aseptate in *C. alboflavescens*) shorter (7.5–22 vs 11–36 μm) ramoconidia. *Cladosporium iranicum* is related with *C. fuscoviride* and *C. alboflavescens*, but can be easily distinguished from them by its larger conidiophores (40–180(–135) μm), with chains of up to 10 conidia in the terminal unbranched part, and a faster growth rate on PDA (56–60 mm after 14 d at 25 °C) (Bensch et al. 2010).

**Figure 5.**
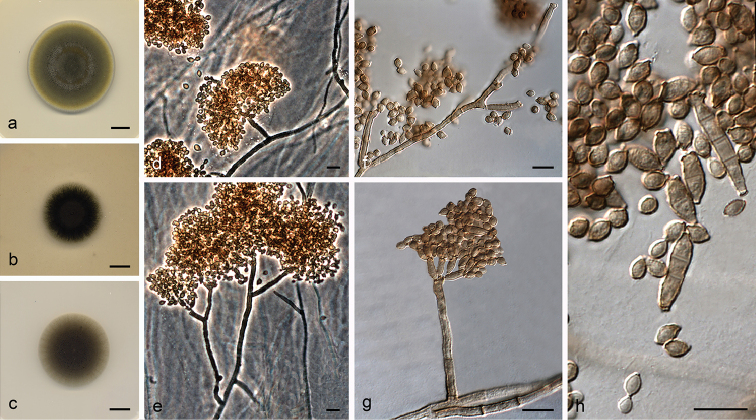
*Cladosporium fuscoviride* (ex-type FMR 16385) **a–c** colonies on PDA, OA and SNA after 14 days at 25 °C **d–g** conidiophores **h** ramoconidia and conidia. Scale bars: 10 mm (**a–c**); 10 μm (**d–h**).

### Cladosporium lentulum

Taxon classificationFungi

Iturrieta-González, Dania García, Gené sp. nov.

FD860513-6747-5319-9B8A-240C77B22F6D

836077

Fig. 6

#### Etymology.

Name refers to its slower growth with respect to the phylogenetically related species (lentus = figuratively slow, with Latin adjectival suffix -ulus = diminutive).

#### Type.

Spain, Catalonia, Tarragona province, Tarragona, unidentified leaf litter, Feb. 2017, *I. Iturrieta-González* (holotype CBS H-24472; cultures ex-type FMR 16288, CBS 146921).

#### Description.

*Mycelium* in vitro superficial and immersed, composed of septate, branched, subhyaline to yellowish brown, smooth to verruculose hyphae, 1–4 μm wide. *Conidiophores* macronematous, arising laterally and terminally from hyphae, septate, erect to slightly flexuous, unbranched, sometimes geniculate at the apex, occasionally branched, up to 406 μm long, 3–4 μm wide, pale brown to brown, smooth to verrucose. *Conidiogenous cells* integrated, terminal and subterminal, cylindrical to subcylindrical, 11–27 × 2–4(–5) μm, bearing up to 5 conidiogenous loci, darkened and refractive. *Ramoconidia* 0(–2)-septate, subcylindrical to cylindrical, 10.5–23 × 2.5–4.5 μm [av. (± SD) 14.2 (± 2.61) × 3.2 (± 0.52)]; pale brown, smooth to verruculose. *Conidia* forming branched chains with up to 5 conidia in the unbranched part of the chain, pale brown, smooth to slightly verruculose, with protuberant, slightly darkened and refractive hila; *small terminal conidia* aseptate obovoidal to ellipsoidal, 4.5–7.5 × 1.5–2.5 μm [av. (± SD) 5.8 (± 0.81)) × 2.7 (± 0.29)]; *intercalary conidia* 0(–1)-septate, ellipsoidal to subcylindrical, 6–10.5 × 2–3 μm [av. (± SD) 8.4 (± 1.31) × 2.3 (± 0.34)]; *secondary ramoconidia* 0(–1)-septate, ellipsoidal to subcylindrical, slightly constricted at septum when present, 7.5–14.5 × 2–3 μm [av. (± SD) 10.5 (± 2.05) × 2.5 (± 0.30)].

#### Culture characteristics

(14 d at 25 °C). Colonies on OA reaching 19–20 mm diam., olive (3F8), flat, velvety, aerial mycelium scarce, margin regular; reverse dark green (30F8) to black. On PDA attaining 28–36 mm diam., dark green (27F8), with a whitish final edge, slightly umbonate, radially folded, velvety, aerial mycelium scarce, margin slightly lobulate; reverse olive brown (4E4), whitish at the edge. On SNA reaching 22–23 mm diam., olive (3F5), flat, slightly dusty, aerial mycelium scarce, margin fimbriate; reverse dark green (30F8) to black.

#### Cardinal temperature for growth.

optimum 20 °C, maximum 30 °C, minimum 5 °C.

#### Distribution.

Spain.

#### Additional specimen examined.

Spain, Catalonia, Tarragona province, Poblet, unidentified herbivore dung, Mar. 2017, *I. Iturrieta-González*, *M. Guevara-Suarez* & *J. Guarro* (FMR 16389).

#### Notes.

Our phylogeny shows *C. lentulum* included in a well-supported terminal clade together with the ex-type strains of *C. exasperatum*, *C. parapenidielloides* and *C. longicatenatum*, three species all described from plant material collected in Australia (Bensch et al. 2010, 2015). However, the genetic distance allows it to be considered a distinct species within the clade (Fig. 1). Phenotypically, *C. lentulum* can be distinguished from its counterparts mainly by its slower growth, especially on OA at 25 °C after 14 d (19–20 mm vs 39–54 mm for *C. exasperatum*, 42–55 mm for *C. parapenidielloides* and 43–54 mm for *C. longicatenatum*). In addition, our new species shows shorter ramoconidia (10.5–23 μm) than *C. exasperatum* and *C. longicatenatum* (19–40 μm and 22–42 μm, respectively); ramoconidia in *C. parapenidielloides* are absent; the conidia in *C. lentulum* are smooth or nearly so, while those of *C. exasperatum* and *C. longicatenatum* possess a unique verruculose-rugose conidial surface ornamentation, especially prominent in the former; and conidiophores in *C. parapenidielloides* are much shorter (up to 67 μm) than those observed in *C. lentulum* (up to 406 μm) (Bensch et al. 2010, 2015).

**Figure 6.**
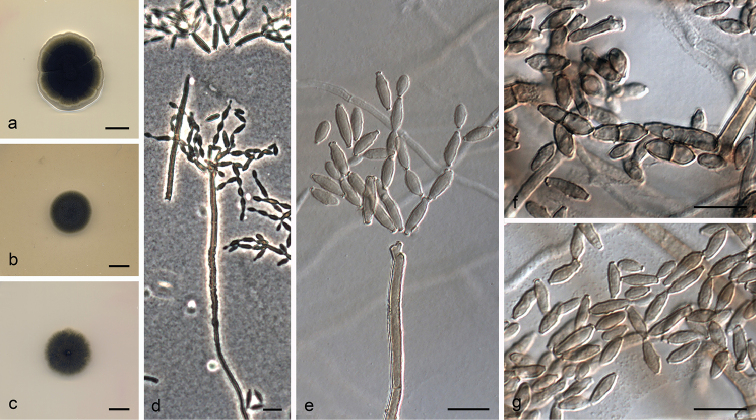
*Cladosporium lentulum* (ex-type FMR 16288) **a–c** colonies on PDA, OA and SNA after 14 days at 25 °C **d–e** conidiophores **f–g** conidia. Scale bars: 10 mm (**a–c**); 10 μm (**d–g**).

### Cladosporium pseudotenellum

Taxon classificationFungi

Iturrieta-González, Dania García, Gené sp. nov.

58A7717F-3728-51CD-A782-33F732E12516

836078

Fig. 7

#### Etymology.

The name refers to “*C. tenellum*”, the closest phylogenetic species.

#### Type.

Spain, Catalonia, Tarragona province, Reus, garden soil, Feb. 2017, *I. Iturrieta-González* (holotype CBS H-24473; cultures ex-type FMR 16231, CBS 146922).

#### Description.

*Mycelium* in vitro superficial and immersed, composed of septate, branched, subhyaline to pale brown, smooth-walled, occasionally tuberculate and with abundant swellings, hyphae, 2–3(–4.5) μm wide. *Conidiophores* macronematous, arising laterally or terminally from hyphae, erect to slightly flexuous, non-nodulose, occasionally geniculate at the apex, septate, unbranched, occasionally branched, up to 146 μm long, 2.5–3 μm wide, pale brown, smooth to slightly verruculose. *Conidiogenous cells* integrated, terminal or intercalary, cylindrical, sometimes geniculate, 15–32 × 2.5–3 μm, with up to five conidiogenous loci, thickened, darkened and refractive, often crowded at the apex. *Ramoconidia* rarely formed, 0(–1)-septate, ellipsoidal to subcylindrical, 9–14.5 × 4–5.5 μm [av. (± SD) 11.6 (± 1.60) × 4.6 (± 0.44)], pale brown, verruculose. *Conidia* forming branched chains, with up to four conidia in the terminal unbranched part, aseptate, pale brown, verruculose to verrucose; *small terminal conidia* subglobose to obovoid, 4–7 × 3–5 μm [av. (± SD) 5.8 (± 0.77) × 3.9 (± 0.60)]; *intercalary conidia* ellipsoidal to limoniform, 6–8.5 × 3–5 μm [av. (± SD) 7.4 (± 0.73) × 3.8 (± 0.50)]; *secondary ramoconidia* 0(–2)-septate, ellipsoidal to subcylindrical, 7–12.5 × 4–5 μm [av. (± SD) 9.6 (± 1.76) × 4.4 (± 0.33)] with 1–3 distal hila.

#### Culture characteristics

(14 d at 25 °C). Colonies on OA reaching 21–22 mm diam., olive (2F8/2F4), flat, velvety, aerial mycelium scarce, margin fimbriate; reverse dark green (30F8) to black. On PDA attaining 29–30 mm diam., olive gray (3E2/3F2), paler at the periphery, radially folded, velvety, aerial mycelium scarce, margin slightly lobate; reverse dark green (30F8) to black. On SNA reaching 21–22 mm diam., olive (2F8), flat, slightly powdery, aerial mycelium scarce, margin fimbriate; reverse dark green (30F8) to black.

#### Cardinal temperature for growth.

Optimum 20 °C, maximum 30 °C, minimum 5 °C.

#### Distribution.

Spain.

#### Notes.

Based on the phylogeny of the *C. herbarum* complex (Fig. 2), *C. pseudotenellum* is closely related with *C. tenellum*, a species originally described from hypersaline water in Israel, later found on *Phyllactinia* sp. (Erysiphaceae), and in indoor air samples collected in the USA (Schubert et al. 2007; Bensch et al. 2012, 2018). Our species differs from *C. tenellum* in the absence of micronematous conidiophores and in having shorter macronematous conidiophores (up to 146 μm vs up to 200 μm), shorter conidiogenous cells (15–32 μm vs 6–40 μm), with few conidiogenous loci (up to five vs up to 10 or more in *C. tenellum*), and shorter ramoconidia (9–14.5 vs up to 32 μm). In addition, terminal and intercalary conidia in *C. pseudotenellum* are aseptate, while those of *C. tenellum* are 0–1(–3)-septate (Schubert et al. 2007; Bensch et al. 2012).

**Figure 7.**
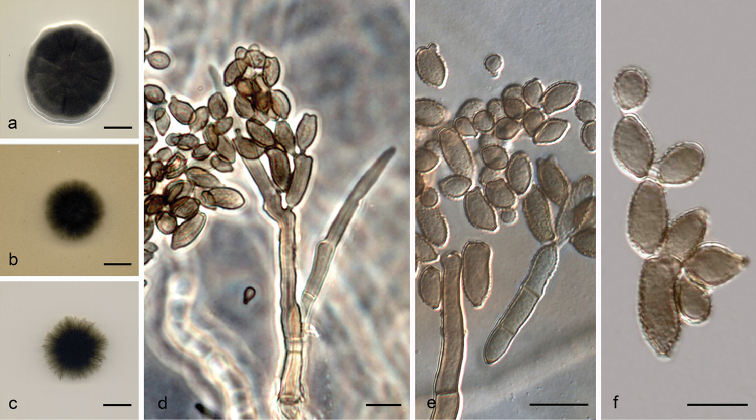
*Cladosporium pseudotenellum* (ex-type FMR 16231) **a–c** colonies on PDA, OA and SNA after 14 days at 25 °C **d–e** conidiophores **f** conidia. Scale bars: 10 mm (**a–c**); 10 μm (**d–f**).

### Cladosporium submersum

Taxon classificationFungi

Iturrieta-González, Dania García, Gené sp. nov.

97217994-1718-5058-9B5F-D6BE9CF41263

836079

Fig. 8

#### Etymology.

Name refers to the aquatic habitat where the substrate (submerged plant material) of the fungus was collected (submersus = submerged, verb in participle, from submergere).

#### Type.

Spain, Catalonia, Tarragona province, Cornudella del Montsant, Siurana’s swamp, submerged plant material, Feb. 2018, *I. Iturrieta-González, E. Carvalho & J. Gené* (holotype CBS H-24474; cultures ex-type FMR 17264, CBS 146923).

#### Description.

*Mycelium* in vitro superficial and immersed, composed of septate, branched, subhyaline to pale brown, smooth-walled to verruculose hyphae, 1–3 μm wide. *Conidiophores* dimorphic, micronematous or macronematous, arising laterally and terminally from hyphae, erect to slightly flexuous, nodulose, geniculate at the apex, septate, unbranched, occasionally branched with small prolongations just below the septum, up to 77 μm long, 3–5 μm wide, pale brown to brown, smooth to verruculose. *Conidiogenous cells* integrated, terminal and intercalary, geniculate, nodulose, 11–28 × 3–6 μm, bearing up to five conidiogenous loci, darkened and refractive. *Ramoconidia* rarely formed, 0(–1)-septate, sometimes constricted at the septum when present, cylindrical to subcylindrical, 10.5–24 × 4.5–7 μm [av. (± SD) 16 (± 3.6) × 6.1 (± 1.03)], pale brown, verruculose to verrucose. *Conidia* forming short branched chains, pale brown, verrucose, occasionally verruculose, with protuberant and slightly darkened hila; *small terminal conidia* aseptate, ovoid to ellipsoidal, 6–12.5 × 3.5–7 μm [av. (± SD) 7.8 (± 1.63) × 4.8 (± 0.79)]; *intercalary conidia* and *secondary ramoconidia* 0–1-septate, ellipsoidal or subcylindrical, 7.5–16 × 4.5–8 μm [av. (± SD) 11 (± 2.18) × 5.7 (± 0.99)].

#### Culture characteristics

(14 d at 25 °C). Colonies on OA reaching 22–23 mm diam., brownish gray to olive brown (4E2/4E4), umbonate, velvety, aerial mycelium scarce, margin slightly irregular and fimbriate; reverse dark green to olive brown (6F8/4E3). On PDA attaining 26–28 mm diam., olive (3F3/1F5), slightly umbonate, radially folded, velvety, aerial mycelium scarce, margin irregularly undulate; reverse dark green (30F9) to black with brownish red (9C6) areas observed between 15 and 20 °C and a white edge. On SNA reaching 21–22 mm diam., olive (3E3), slightly umbonate, loosely cottony, margin fimbriate; reverse dark olive brown to golden gray (3E3/4C2).

#### Cardinal temperature for growth.

Optimum 20 °C, maximum 35 °C, minimum 5 °C.

#### Distribution.

Spain.

#### Notes.

*Cladosporium submersum* is related to *C. subcinereum*, and morphologically differentiated by having shorter conidiophores (up to 77 μm vs up to 140 μm), shorter conidiogenous cells (11–28 vs 16–38 μm), shorter ramoconidia (10.5–24 vs 19–59 μm), and longer terminal conidia (6–12.5 vs 5–7 μm), which are ovoid to ellipsoidal in our species and globose to subglobose in *C. subcinereum* (Sandoval-Denis et al. 2016). In addition, *C. submersum* exhibited a colony reverse on PDA with brownish red areas, a feature that is absent in *C. subcinereum*.

**Figure 8.**
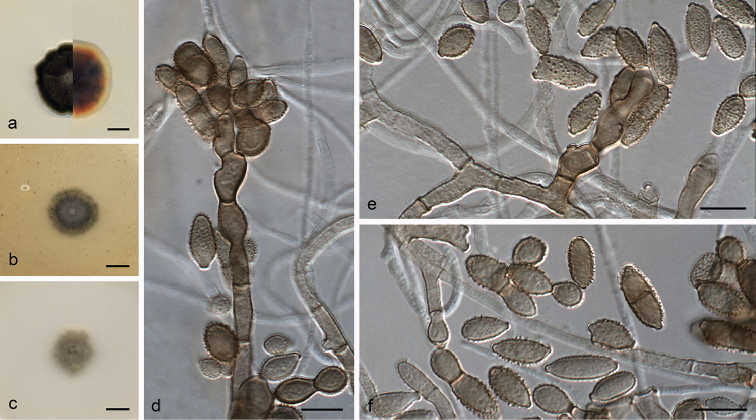
*Cladosporium submersum* (ex-type FMR 16264) **a–c** colonies on PDA (front at 25 °C and reverse at 20 °C), OA and SNA at 25 °C after 14 days **d, e** conidiophores and conidia **f** conidia. Scale bars: 10 mm (**a–c**); 10 μm (**d–f**).

## Discussion

*Cladosporium* is a well-delineated genus, the taxonomic structure and phylogenetic relationships of its species have been investigated in several studies over the last decade, so far giving rise to a genus of more than two hundred well-established species (Zalar et al. 2007; Bensch et al. 2010, 2012, 2018; Sandoval-Denis et al. 2016; Marin-Felix et al. 2017; Crous et al. 2009, 2019; Jayasiri et al. 2019). However, this species number will continue to expand through the study of soil, which is a proven pool of fungal species that remains undescribed, and other substrates poorly investigated by molecular tools for fungal diversity (Tedersoo et al. 2017; Hyde et al. 2018). In this context, a set of *Cladosporium* isolates were obtained in pure culture from samples of soil, dung from different herbivorous animals, and plant debris collected during a survey of microfungi in various Spanish locations. Using the molecular approach for species delineation in *Cladosporium* (Bensch et al. 2012; Marin-Felix et al. 2017), eight of those isolates represented six novel lineages for the genus which are proposed as *C. caprifimosum*, *C. coprophilum*, *C. fuscoviride*, *C. lentulum*, *C. pseudotenellum* and *C. submersum*. Of note is that almost all the specimens in the present study (7/8) were isolated directly from the natural substratum incubated in moist chambers or from baiting technique plates. Although *Cladosporium* isolates are commonly detected by plating methods, the slow growth rate or the low spore concentration of some cladosporium-like fungi compared to other fungi present in a given substrate is probably a handicap to detection and/or isolation of uncommon *Cladosporium* species. Therefore, as recommended by Crous (1998) for similar fungi, techniques based on fungal isolation directly from the natural substratum should be considered a choice for future studies of *Cladosporium* species diversity.

To our knowledge, *Cladosporium* species as dung inhabiting fungi have been reported in a very few studies, *C. cladosporioides* and *C. herbarum* being the most reported species (Bell 1975; Seifert et al. 1983; Jeamjitt et al. 2006; Masunga et al. 2006; Piontelli et al. 2006; Simões-Calaça et al. 2014; Thilagam et al. 2015). However, in all those studies, fungal identification was based exclusively on morphological features. Only *C. herbarum* has been reported recently from crown droppings and identified molecularly, but using only the ITS barcode (Torbati et al. 2016). In our case, the three new species isolated on herbivore dung (i.e., *C. caprifimosum*, *C. coprophilum*, and *C. lentulum*) showed the typical morphological features attributed to the *C. cladosporioides* species complex. However, their identifications would have been difficult with morphological features alone, even with the analysis of their ITS sequences (Table 2) since they are identical under the universal barcode for fungi as reported in previous studies for many other *Cladosporium* species (Bensch et al. 2010, 2012; Marin-Felix et al. 2017). Therefore, only sequence analysis with *act* and *tef1* will allow us to know the real diversity of *Cladosporium* species from this understudied substrate by molecular tools.

Although no temperature studies have been systematically applied to characterize most *Cladosporium* species (Bensch et al. 2012, 2015, 2018), we agree with Ma et al. (2017) that cardinal temperatures for growth can help to differentiate certain species in their respective complexes. While species in the *C. sphaerospermum* complex show a maximum temperature for growth of no more than 30–32 °C, *C. halotolerans* was able to grow at 35 °C (Sandoval-Denis et al. 2015). Similarly, although most species of the *C. cladosporioides* complex do not tolerate high temperatures, *C. angulosum*, *C. angustisporum*, *C. anthropophilum*, *C. flavovirens*, *C. funiculosum*, *C. pseudocladosporioides*, *C. subuliforme* and *C. tenuissimum* were able to grow at 35 °C (Sandoval-Denis et al. 2015, 2016). To date, no member of the *C. herbarum* complex was found to be able to grow above 30 °C; however, one of the novel species of the complex described here, *C. submersum*, had a maximum growth at 35 °C. On the contrary, the recently described species *C. neopsychrotolerans* and *C. tianshanense* from the complex *C. cladosporioides* and *C. psychrotolerans* from the complex *C. sphaerospermum* showed a psychrophilic behavior (Zalar et al. 2007; Ma et al. 2017), demonstrating in part the ability of *Cladosporium* species to adapt to different environmental conditions.

## Supplementary Material

XML Treatment for Cladosporium caprifimosum

XML Treatment for Cladosporium coprophilum

XML Treatment for Cladosporium fuscoviride

XML Treatment for Cladosporium lentulum

XML Treatment for Cladosporium pseudotenellum

XML Treatment for Cladosporium submersum

## Supplementary materials

Supplementary material 1 Table S1 Data type Species name and strains data Explanation note Species, strain information and GenBank accession numbers of the sequences included in the phylogenetic analyses. https://binary.pensoft.net/file/494244This dataset is made available under the Open Database License (http://opendatacommons.org/licenses/odbl/1.0/). The Open Database License (ODbL) is a license agreement intended to allow users to freely share, modify, and use this Dataset while maintaining this same freedom for others, provided that the original source and author(s) are credited.Isabel Iturrieta-González, Dania García, Josepa Gené
