# Supplementary material for: Novel species of Cladosporium from environmental sources in Spain
Source: MycoKeys. 2021 Jan 5;77:1–25. doi: 10.3897/mycokeys.77.60862 (PMC7803722; doi:10.3897/mycokeys.77.60862)
Supplement: Supplementary material 1 — Table S1 [file mycokeys-77-001-s001.docx]

|  |
| --- |

**Supplementary material**

**Table S1.** Species, strains and GenBank accession number of the sequences included in this study.

| **Species** | **Strain no.^1^** | **Substrate^2^** | **GenBank nucleotide accession no. for^3^**: | | | **References** |
| --- | --- | --- | --- | --- | --- | --- |
|  |  |  | **ITS** | ***act*** | ***tef1*** |  |
| *Cercospora beticola* | CBS 116456 | *Beta vulgaris* | NR121315 | AY840458 | AY840494 | Bensch et al. 2012 |
| *Cladosporium acalyphae* | CBS 125982^T^ | *Acalypha australis* | HM147994 | HM148481 | HM148235 | Bensch et al. 2010 |
| *C. aerium* | CBS 143356^T^ | Indoor air | MF472897 | MF473747 | MF473324 | Bensch et al. 2018 |
|  | DTO 323-G7 | Indoor air | MF472899 | MF473749 | MF473326 | Bensch et al. 2018 |
| *C. aggregatocicatricatum* | CBS 140493^T^ | Culture contaminant | KT600448 | KT600645 | KT600547 | Bensch et al. 2015 |
| *C. alboflavescens* | CBS 140690^T^ | Animal BAL | LN834420 | LN834604 | LN834516 | Sandoval-Denis et al. 2016 |
| *C. allii* | CBS 101.81 | Velvet spots on *Allium porrum* | JN906977 | JN906996 | JN906983 | Bensch et al. 2012 |
| *C. allicinum* | CBS 121624^T^ | *Hordeum vulgare* | EF679350 | EF679502 | EF679425 | Schubert et al. 2007 |
|  | UTHSC DI-13-176 | Human skin | LN834354 | LN834538 | LN834450 | Sandoval-Denis et al. 2015 |
| *C. angulosum* | CBS 140692^T^ | Human BAL | LN834425 | LN834609 | LN834521 | Sandoval-Denis et al. 2016 |
| *C. angustiherbarum* | CBS 140479^T^ | *Pinus ponderosa* | KT600378 | KT600574 | KT600475 | Bensch et al. 2015 |
| *C. angustisporum* | CBS 125983^T^ | *Alloxylon wickhamii* | HM147995 | HM148482 | HM148236 | Bensch et al. 2010 |
| *C. angustiterminale* | CBS 140480^T^ | *Banksia grandis* | KT600379 | KT600575 | KT600476 | Bensch et al. 2015 |
| *C. antarcticum* | CBS 690.92^T^ | *Caloplaca regalis* | EF679334 | EF679484 | EF679405 | Schubert et al. 2007 |
| *C. anthropophilum* | CBS 140685^T^ | Human BAL | LN834437 | LN834621 | LN834533 | Sandoval-Denis et al. 2016 |
|  | CBS 674.82 | Seed of *Gossypium* sp. | HM148014 | HM148501 | HM148255 | Bensch et al. 2010 |
| *C. arthropodii* | CBS 124043^ET^ | *Arthropodium cirratum* | JN906979 | JN906998 | JN906985 | Bensch et al. 2012 |
| *C. asperulatum* | CBS 126339 | Leaf litter of *Eucalyptus* sp. | HM147997 | HM148484 | HM148238 | Bensch et al. 2010 |
|  | CBS 126340^T^ | *Protea susannae* | HM147998 | HM148485 | HM148239 | Bensch et al. 2010 |
| *C. australiense* | CBS 125984^T^ | *Eucalyptus moluccana* | HM147999 | HM148486 | HM148240 | Bensch et al. 2010 |
| *C. austroafricanum* | CBS 140481^T^ | Leaf litter | KT600381 | KT600577 | KT600478 | Bensch et al. 2015 |
| *C. basiinflatum* | CBS 822.84^T^ | *Hordeum vulgare* | HM148000 | HM148487 | HM148241 | Bensch et al. 2015 |
| ***C.* *caprifimosum*** | **FMR 16532^T^**  **(CBS 146918)** | **Goat dung** | **LR813198** | **LR813205** | **LR813210** | **Present study** |
| *C. chalastosporoides* | CBS 125985^T^ | Fruiting bodies of *Teratosphaeria proteae-arboreae* | HM148001 | HM148488 | HM148242 | Bensch et al. 2010 |
| *C. chasmanthicola* | CBS 142612^T^ | Leaf spots of *Chasmanthe aethiopica* | KY646221 | KY646224 | KY646227 | Marin-Felix et al. 2017 |
| *C. chubutense* | CBS 124457^T^ | *Pinus ponderosa* | FJ936158 | FJ936165 | FJ936161 | Schubert et al. 2009 |
| *C. cladosporioides* | CBS 112388^T^ | Air of indoor environment | HM148003 | HM148490 | HM148244 | Bensch et al. 2010 |
|  | CBS 113738 | Grape bud | HM148004 | HM148491 | HM148245 | Bensch et al. 2010 |
| *C. colocasiae* | CBS 386.64^T^ | *Colocasia esculenta* | HM148067 | HM148555 | HM148310 | Bensch et al. 2010 |
|  | CBS 119542 | *Colocasia esculenta* | HM148066 | HM148554 | HM148309 | Bensch et al. 2010 |
| *C. colombiae* | CBS 274.80B^T^ | *Cortaderia* sp. | FJ936159 | FJ936166 | FJ936163 | Schubert et al. 2009 |
| ***C.* *coprophilum*** | **FMR 16101** | **Unidentified herbivore dung** | **LR813199** | **LR813204** | **LR813211** | **Present study** |
|  | **FMR 16164^T^**  **(CBS 144919)** | **Unidentified herbivore dung** | **LR813201** | **LR813207** | **LR813213** | **Present study** |
| *C. crousii* | CBS 140686^T^ | Human BAL | LN834431 | LN834615 | LN834527 | Sandoval-Denis et al. 2016 |
| *C. cucumerinum* | CBS 171.52^T^ | *Cucumis sativus* | HM148072 | HM148561 | HM148316 | Bensch et al. 2010 |
|  | CBS 176.54 | *Cucumis sativus* | HM148078 | HM148567 | HM148322 | Bensch et al. 2010 |
| *C. delicatulum* | CBS 126344^T^ | Leaves of *Tilia cordata* | HM148081 | HM148570 | HM148325 | Bensch et al. 2010 |
|  | CBS 126342 | Indoor air | HM148079 | HM148568 | HM148323 | Bensch et al. 2010 |
| *C. echinulatum* | CBS 123191 | *Dianthus barbatus* | JN906980 | JN906999 | JN906987 | Bensch et al. 2012 |
| *C. europaeum* | CBS 116744 | Leaves of *Acer pseudoplatanus* | HM148053 | HM148540 | HM148294 | Bensch et al. 2018 |
|  | CBS 134914^T^ | Indoor building material | HM148056 | HM148543 | HM148298 | Bensch et al. 2018 |
| *C. exasperatum* | CBS 125986^T^ | *Eucalyptus tintinnans* | HM148090 | HM148579 | HM148334 | Bensch et al. 2010 |
| *C. exile* | CBS 125987^T^ | Chasmothecia of *Phyllactinia guttata* | HM148091 | HM148580 | HM148335 | Bensch et al. 2010 |
| *C. fildesense* | ChFC-554^T^ | Unidentified marine sponge | JX845290 | MN233632 | MN233633 | Crous et al. 2019 |
| *C. flavovirens* | CBS 140462^T^ | Human toenail | LN834440 | LN834624 | LN834536 | Sandoval-Denis et al. 2016 |
| *C. flabelliforme* | CBS 126345^T^ | *Melaleuca cajuputi* | HM148092 | HM148581 | HM148336 | Bensch et al. 2010 |
| *C. floccosum* | CBS 140463^T^ | Humans ethmoid sinus | LN834416 | LN834600 | LN834512 | Sandoval-Denis et al. 2016 |
| *C. funiculosum* | CBS 122128 | *Ficus carica* | HM148093 | HM148582 | HM148337 | Bensch et al. 2010 |
|  | CBS 122129^T^ | *Vigna umbellata* | HM148094 | HM148583 | HM148338 | Bensch et al. 2010 |
| ***C.* *fuscoviride*** | **FMR 16385^T^**  **(CBS 146920)** | **Garden soil** | **LR813200** | **LR813206** | **LR813212** | **Present study** |
| *C. gamsianum* | CBS 125989^T^ | *Strelitzia* sp. | HM148095 | HM148584 | HM148339 | Bensch et al. 2010 |
| *C. globisporum* | CBS 812.96^T^ | Meat stamp | HM148096 | HM148585 | HM148340 | Bensch et al. 2010 |
| *C. grevilleae* | CBS 114271^T^ | Leaves of *Grevillea* sp. | JF770450 | JF770473 | JF770472 | Crous et al. 2011 |
| *C. herbaroides* | CBS 121626^T^ | Hypersaline water | EF679357 | EF679509 | EF679432 | Schubert et al. 2007 |
| *C. herbarum* | CBS 121621^ET^ | *Hordeum vulgare* | EF679363 | EF679516 | EF679440 | Schubert et al. 2007 |
| *C. hillianum* | CPC 15458 | Leaf of *Typha orientalis* | HM148098 | HM148587 | HM148342 | Bensch et al. 2010 |
|  | CBS 125988^T^ | Leaf of *Typha orientalis* | HM148097 | HM148586 | HM148341 | Bensch et al. 2010 |
| *C. inversicolor* | CBS 401.80^T^ | Leaf of *Triticum aestivum* | HM148101 | HM148590 | HM148345 | Bensch et al. 2010 |
|  | CBS 143.65 | Leaf of *Tilia* sp. | HM148100 | HM148589 | HM148344 | Bensch et al. 2010 |
| *C. ipereniae* | CPC 16855 | *Arctostaphylos pallida* | KT600395 | KT600590 | KT600492 | Bensch et al. 2015 |
|  | CBS 140483^T^ | *Puya* sp. | KT600394 | KT600589 | KT600491 | Bensch et al. 2015 |
| *C. iranicum* | CBS 126346^T^ | Leaf of *Citrus sinensis* | HM148110 | HM148599 | HM148354 | Bensch et al. 2010 |
| *C. iridis* | CBS 138.40^ET^ | Leaves of *Iris* sp. | EF679370 | EF679523 | EF679447 | Schubert et al. 2007 |
| *C. kenpeggii* | CBS 142613^T^ | Leaves of *Passiflora edulis* | KY646222 | KY646225 | KY646228 | Marin-Felix et al. 2017 |
| ***C.* *lentulum*** | **FMR 16288^T^**  **(CBS 146921)** | **Unidentified leaf litter** | **LR813203** | **LR813209** | **LR813215** | **Present study** |
|  | **FMR 16389** | **Unidentified herbivore dung** | **LR813202** | **LR813208** | **LR813214** | **Present study** |
| *C. limoniforme* | CBS 140484^T^ | *Musa acuminata* | KT600397 | KT600592 | KT600494 | Bensch et al. 2015 |
|  | CBS 113737 | Grape berry | KT600396 | KT600591 | KT600493 | Bensch et al. 2015 |
| *C. licheniphilum* | CBS 125990^ET^ | *Phaeophyscia orbicularis* and *Physcia* sp. | HM148111 | HM148600 | HM148355 | Bensch et al. 2010 |
| *C. longicatenatum* | CBS 140485^T^ | Unknown plant | KT600403 | KT600598 | KT600500 | Bensch et al. 2015 |
| *C. longissimum* | CBS 300.96 ^T^ | Soil along coral reef coast | DQ780352 | EF101385 | EU570259 | Zalar et al. 2007, Dugan et al. 2008 |
| *C. lycoperdinum* | CBS 126347 | Galls of *Apiosporina morbosa* | HM148112 | HM148601 | HM148356 | Bensch et al. 2010 |
|  | CBS 574.78C | *Aureobasidium caulivorum* | HM148115 | HM148604 | HM148359 | Bensch et al. 2010 |
| *C. macrocarpum* | CBS 121623^NT^ | *Spinacia oleracea* | EF679375 | EF679529 | EF679453 | Schubert et al. 2007 |
|  | UTHSC DI-13-191 | Human face | LN834379 | LN834563 | LN834475 | Sandoval-Denis et al. 2015 |
| *C. magnoliigena* | MFLUCC 18–1559 ^T^ | Cone of *Magnolia grandiflora* | MK347813 | - | MK340864 | Jayasiri et al. 2019 |
|  | MFLUCC 18–1557 | Cone of *Magnolia grandiflora* | MK347811 | - | MK340862 | Jayasiri et al. 2019 |
| *C. montecillanum* | CBS 140486^T^ | Pine needles | KT600406 | KT600602 | KT600504 | Bensch et al. 2015 |
|  | CPC 15605 | *Taraxacum* sp. | KT600407 | KT600603 | KT600505 | Bensch et al. 2015 |
| *C. myrtacearum* | CBS 126349 | *Eucalyptus placita* | HM148116 | HM148605 | HM148360 | Bensch et al. 2010 |
|  | CBS 126350^T^ | *Corymbia foelscheana* | HM148117 | HM148606 | HM148361 | Bensch et al. 2010 |
| *C. needhamense* | CBS 143359^T^ | Indoor (office) air sample | MF473142 | MF473991 | MF473570 | Bensch et al. 2018 |
| *C. neerlandicum* | CBS 143360^T^ | Swab sample | KP701887 | KP702010 | KP701764 | Segers et al. 2015 |
| *C. neopsychrotolerans* | CGMCC 3.18031^T^ | Soil of *Saussurea involucrata* | KX938383 | KX938366 | KX938400 | Ma et al. 2017 |
|  | CGMCC 3.18032 | Soil of *Saussurea involucrata* | KX938384 | KX938367 | KX938401 | Ma et al. 2017 |
| *C. ossifragi* | CBS 842.91^ET^ | Green leaf of *Narthecium ossifragum* | EF679381 | EF679535 | EF679459 | Schubert et al. 2007 |
| *C. oxysporum* | CBS 125991 | Soil, near the terracotta army | HM148118 | HM148607 | HM148362 | Bensch et al. 2010 |
|  | CBS 126351 | Indoor air | HM148119 | HM148608 | HM148363 | Bensch et al. 2010 |
| *C. paracladosporioides* | CBS 171.54^T^ | Unknown | HM148120 | HM148609 | HM148364 | Bensch et al. 2010 |
| *C. paralimoniforme* | CGMCC 3.18103^T^ | Soil | KX938392 | KX938375 | KX938409 | Ma et al. 2017 |
|  | CGMCC 3.18104 | Soil | KX938393 | KX938376 | KX938410 | Ma et al. 2017 |
| *C. parapenidielloides* | CBS 140487^T^ | *Eucalyptus* sp. | KT600410 | KT600606 | KT600508 | Bensch et al. 2015 |
| *C. parasubtilissimum* | CBS 143361^T^ | Indoor (bathroom) air | MF473170 | MF474018 | MF473593 | Bensch et al. 2018 |
|  | CPC 22396 | Indoor (recreational vehicle) air | MF473171 | MF474019 | MF473594 | Bensch et al. 2018 |
| *C. perangustum* | CBS 125996^T^ | *Cussonia* sp. | HM148121 | HM148610 | HM148365 | Bensch et al. 2010 |
| *C. phaenocomae* | CBS 128769^T^ | *Phaenocoma prolifera* | JF499837 | JF499881 | JF499875 | Crous & Groenewald 2011 |
| *C. phlei* | CBS 358.69^ET^ | *Phleum pratense* | JN906981 | JN907000 | JN906991 | Bensch et al. 2012 |
| *C. phyllactiniicola* | CBS 126352^T^ | Chasmothecia of *Phyllactinia guttata* | HM148150 | HM148639 | HM148394 | Bensch et al. 2010 |
|  | CBS 126355 | Chasmothecia of *Phyllactinia guttata* | HM148153 | HM148642 | HM148397 | Bensch et al. 2010 |
| *C. phyllophilum* | CPC 13873 | *Teratosphaeria proteae-arboreae* on *Protea arborea* | HM148155 | HM148644 | HM148399 | Bensch et al. 2010 |
|  | CBS 125992^ET^ | *Taphrina* sp. on *Prunus cerasus* | HM148154 | HM148643 | HM148398 | Bensch et al. 2010 |
| *C. pini-ponderosae* | CBS 124456^T^ | *Pinus ponderosa* | FJ936160 | FJ936167 | FJ936164 | Schubert et al. 2009 |
| *C. prolongatum* | CGMCC 3.18035 | Soil of *Populus euphratica* | KX938395 | KX938378 | KX938412 | Ma et al. 2017 |
|  | CGMCC 3.18036^T^ | Soil of *Populus euphratica* | KX938394 | KX938377 | KX938411 | Ma et al. 2017 |
| *C. pseudiridis* | CBS 116463^T^ | Leaf lesions on *Iris* sp. | EF679383 | EF679537 | EF679461 | Schubert et al. 2007 |
| ***C.* *pseudotenellum*** | **FMR 16231^T^**  **(CBS 146922)** | **Garden soil** | **LR813145** | **LR813146** | **LR813196** | **Present study** |
| *C. pseudochalastosporoides* | CBS 140490^T^ | Pine needles | KT600415 | KT600611 | KT600513 | Bensch et al. 2015 |
| *C. pseudocladosporioides* | CBS 125993^T^ | Outside air | HM148158 | HM148647 | HM148402 | Bensch et al. 2015 |
| *C. puyae* | CBS 274.80A^T^ | *Puya goudotiana* | KT600418 | KT600614 | KT600516 | Bensch et al. 2015 |
| *C. ramotenellum* | CBS 121628^T^ | Hypersaline water | EF679384 | EF679538 | EF679462 | Schubert et al. 2007 |
| *C. rectoides* | CBS 125994^T^ | *Vitis flexuosa* | HM148193 | HM148683 | HM148438 | Bensch et al. 2010 |
|  | CBS 126357 | *Plectranthus* sp. | HM148194 | HM148684 | HM148439 | Bensch et al. 2010 |
| *C. rhusicola* | CBS 140492^T^ | *Rhus* sp. | KT600440 | KT600637 | KT600539 | Bensch et al. 2015 |
| *C. rugulovarians* | CBS 140495^T^ | Leaf of unidentified *Poaceae* | KT600459 | KT600656 | KT600558 | Bensch et al. 2015 |
| *C. scabrellum* | CBS 126358^T^ | *Ruscus hypoglossum* | HM148195 | HM148685 | HM148440 | Bensch et al. 2010 |
| *C. silenes* | CBS 109082^T^ | *Silene maritima* | EF679354 | EF679506 | EF679429 | Schubert et al. 2007 |
| *C. sinense* | CBS 143363^T^ | Indoor air | MF473252 | MF474102 | MF473675 | Bensch et al. 2018 |
| *C. sinuatum* | CGMCC 3.18096^T^ | Alpine soil | KX938385 | KX938368 | KX938402 | Ma et al. 2017 |
|  | CGMCC 3.18097 | Alpine soil | KX938386 | KX938369 | KX938403 | Ma et al. 2017 |
| *C. sinuosum* | CBS 121629^T^ | *Fuchsia excorticata* | EF679386 | EF679540 | EF679464 | Schubert et al. 2007 |
|  | CBS 393.68 | Air | KT600442 | KT600639 | KT600541 | Bensch et al. 2015 |
| *C. soldanellae* | CBS 132186^NT^ | *Soldanella alpina* | JN906982 | JN907001 | JN906994 | Bensch et al. 2012 |
| *C. sphaerospermum* | CBS 193.54 | Human nails | DQ780343 | EU570269 | EU570261 | Zalar et al. 2007, Bensch et al. 2012 |
| *C. spinulosum* | CBS 119907^T^ | Hypersaline water | EF679388 | EF679542 | EF679466 | Schubert et al. 2007 |
| *C. subcinereum* | CBS 140465^T^ | Human sputum | LN834433 | LN834617 | LN834529 | Sandoval-Denis et al. 2016 |
| *C. subinflatum* | CBS 121630^T^ | Hypersaline water | EF679389 | EF679543 | EF679467 | Schubert et al. 2007 |
|  | UTHSC DI-13-189 | Human toenail | LN834391 | LN834575 | LN834487 | Sandoval-Denis et al. 2015 |
| ***C.* *submersum*** | **FMR 17264^T^**  **(CBS 146923)** | **Submerged plant material** | **LR813144** | **LR813195** | **LR813197** | **Present study** |
| *C. subtilissimum* | CBS 113754^T^ | Grape berry | EF679397 | EF679551 | EF679475 | Schubert et al. 2007 |
|  | CBS 113753 | Bing cherry fruit | EF679396 | EF679550 | EF679474 | Schubert et al. 2007 |
| *C. subuliforme* | CBS 126500^T^ | *Chamaedorea metallica* | HM148196 | HM148686 | HM148441 | Bensch et al. 2010 |
|  | CPC 15833 | *Citrus* sp. | KT600453 | KT600650 | KT600552 | Bensch et al. 2015 |
| *C. tenellum* | CBS 121634^T^ | Hypersaline water | EF679401 | EF679555 | EF679479 | Schubert et al. 2007 |
|  | CPC 11813 | *Phyllactinia* sp. on leaves of *Corylus* sp. | EF679399 | EF679553 | EF679477 | Schubert et al. 2007 |
|  | CPC 12051 | Hypersaline water | EF679400 | EF679554 | EF679478 | Schubert et al. 2007 |
|  | CPC 22290 | Indoor air | MF473278 | MF474128 | MF473701 | Bensch et al. 2018 |
|  | CPC 22291 | Indoor air | MF473279 | MF474129 | MF473702 | Bensch et al. 2018 |
|  | CPC 22410 | Indoor air | MF473280 | MF474130 | MF473703 | Bensch et al. 2018 |
|  | DTO 127-D7 | Air | KP701932 | KP702054 | KP701809 | Segers et al. 2015 |
| *C. tenuissimum* | CBS 125995^ET^ | *Lagerstroemia* sp. | HM148197 | HM148687 | HM148442 | Bensch et al. 2010 |
| *C. tianshanense* | CGMCC 3.18033^T^ | Soil of *Saussurea involucrata* | KX938381 | KX938364 | KX938398 | Ma et al. 2017 |
|  | CGMCC 3.18034 | Soil of *Saussurea involucrata* | KX938382 | KX938365 | KX938399 | Ma et al. 2017 |
| *C. tuberosum* | CBS 140693^T^ | Human nasal biopsy | LN834417 | LN834601 | LN834513 | Sandoval-Denis et al. 2016 |
|  | UTHSC DI-13-219 | Human foot | LN834419 | LN834603 | LN834515 | Sandoval-Denis et al. 2016 |
| *C. uwebraunianum* | CBS 143365^T^ | Indoor (archive) air | MF473306 | MF474156 | MF473729 | Bensch et al. 2018 |
|  | DTO 072-C8 | Indoor (archive) air | KP701873 | KP701996 | KP701750 | Bensch et al. 2018 |
| *C. variabile* | CBS 121635^ET^ | *Spinacia oleracea* | EF679402 | EF679556 | EF679480 | Schubert et al. 2007 |
| *C. varians* | CBS 126360 | *Ulmus* sp. | HM148222 | HM148713 | HM148468 | Bensch et al. 2010 |
|  | CBS 126362^T^ | *Catalpa bungei* | HM148224 | HM148715 | HM148470 | Bensch et al. 2010 |
| *C. versiforme* | CBS 140491^T^ | *Hordeum* sp. | KT600417 | KT600613 | KT600515 | Bensch et al. 2015 |
| *C. verruculosum* | CGMCC 3.18099^T^ | Alpine soil | KX938388 | KX938371 | KX938405 | Ma et al. 2017 |
|  | CGMCC 3.18100 | Alpine soil | KX938389 | KX938372 | KX938406 | Ma et al. 2017 |
| *C. verrucocladosporioides* | CBS 126363^T^ | *Rhus chinensis* | HM148226 | HM148717 | HM148472 | Bensch et al. 2010 |
| *C. vicinum* | CBS 143366^T^ | Indoor air | MF473311 | MF474161 | MF473734 | Bensch et al. 2018 |
|  | CBS 306.84 | Urediniospores of *Puccinia allii* | HM148057 | HM148544 | HM148299 | Bensch et al. 2018 |
| *C. welwitschiicola* | CBS 142614^T^ | Dead leaf of *Welwitschia mirabilis* | KY646223 | KY646226 | KY646229 | Marin-Felix et al. 2017 |
| *C. westerdijkiae* | CBS 113746^T^ | Bing cherry fruits | HM148061 | HM148548 | HM148303 | Bensch et al. 2018 |
|  | CPC 10150 | *Fatoua villosa* | HM148062 | HM148549 | HM148304 | Bensch et al. 2018 |
| *C. wyomingense* | CBS 143367^T^ | Indoor (living room) air | MF473315 | MF474165 | MF473738 | Bensch et al. 2018 |
| *C. xanthochromaticum* | CBS 140691^T^ | Human BAL | LN834415 | LN834599 | LN834511 | Sandoval-Denis et al. 2016 |
| *C. xylophilum* | CBS 113749 | Bing cherry fruits | HM148228 | HM148719 | HM148474 | Bensch et al. 2010 |
|  | CBS 125997^T^ | Dead wood of *Picea abies* | HM148230 | HM148721 | HM148476 | Bensch et al. 2010 |

^1^ CBS: Culture collection of the Westerdijk Fungal Biodiversity Institute, Utrecht, the Netherlands; CGMCC: China General Microbiological Culture Collection Center, Institute of Microbiology, Chinese Academy of Sciences, Beijing, China; ChFC: Chilean Fungal Collection, Chile; CPC: Culture collection of Pedro Crous, housed at CBS; DTO: Working collection of Jos Houbraken housed at CBS; FMR: Facultat de Medicina i Ciències de la Salut, Reus, Spain; MFLUCC: Mae Fah Luang University Culture Collection, Chiang Rai, Thailand; UTHSC: Fungus Testing Laboratory at the University of Texas Health Science Center, San Antonio, TX, USA.^T, ET, NT^, indicate ex-type, ex-epitype and ex-neotype strains, respectively. ^2^ BAL: bronchoalveolar lavage fluid. ^3^ ITS: Internal transcribed spacer regions of the rDNA and 5.8S region; *act*: partial actin gene; *tef1*: partial translation elongation factor 1-alpha gene; Sequences newly generated in this study and novel species proposed are indicated in bold.

**References in Table S1 not included in the main text**

Crous PW, Groenewald JZ (2011) Why everlastings don’t last. Persoonia 26: 70–84.

Crous PW, Tanaka K, Summerell BA, Groenewald JZ (2011) Additions to the *Mycosphaerella* complex. IMA Fungus 2: 49–64.

Dugan FM, Braun U, Groenewald JZ, Crous PW (2008) Morphological plasticity in *Cladosporium sphaerospermum*. Persoonia 21: 9–16.

Segers FJJ, Meijer M, Houbraken J, Samson RA, Wösten HAB, et al. (2015) Xerotolerant *Cladosporium sphaerospermum* are predominant on indoor surfaces compared to other *Cladosporium* species. Plos One 10(12): e0145415. doi:10.1371/journal.pone.0145415.
